# Evaluation of Commercially Available Anti–Dengue Virus Immunoglobulin M Tests

**DOI:** 10.3201/eid1503.080923

**Published:** 2009-03

**Authors:** Elizabeth A. Hunsperger, Sutee Yoksan, Philippe Buchy, Vinh Chau Nguyen, Shamala D. Sekaran, Delia A. Enria, Jose L. Pelegrino, Susana Vázquez, Harvey Artsob, Michael Drebot, Duane J. Gubler, Scott B. Halstead, María G. Guzmán, Harold S. Margolis, Carl-Michael Nathanson, Nidia R. Rizzo Lic, Kovi E. Bessoff, Srisakul Kliks, Rosanna W. Peeling

**Affiliations:** Centers for Disease Control and Prevention, San Juan, Puerto Rico, USA (E.A. Hunsperger, K.E. Bessoff); Mahidol University, Bangkok, Thailand (S. Yoksan); Institut Pasteur, Phnom Penh, Cambodia (P. Buchy); Cho Quan Hospital, Ho Chi Minh City, Vietnam (V.C. Nguyen); University of Malaya, Kuala Lumpur, Malaysia (S.D. Sekaran); Instituto Nacional Enfermedades Virales Humanas Dr. Julio I. Maiztegui, Buenos Aires, Argentina (D.A. Enria); Instituto Medicina Tropical Pedro Kouri, Havana, Cuba (S. Vázquez, M.G. Guzmán, J.L. Pelegrino); Public Health Agency of Canada, Winnipeg, Manitoba, Canada (H. Artsob, M. Drebot); University of Hawaii, Honolulu, Hawaii, USA (D.J. Gubler); Pediatric Dengue Vaccine Initiative, Seoul, South Korea (S.B. Halstead, H.S. Margolis, S. Kliks); World Health Organization, Geneva, Switzerland (C.-M. Nathanson, N.R. Rizzo, R.W. Peeling)

**Keywords:** Dengue fever, immunoglobulin M, ELISA, diagnostic test, dispatch

## Abstract

Anti–dengue virus immunoglobulin M kits were evaluated. Test sensitivities were 21%–99% and specificities were 77%–98% compared with reference ELISAs. False-positive results were found for patients with malaria or past dengue infections. Three ELISAs showing strong agreement with reference ELISAs will be included in the World Health Organization Bulk Procurement Scheme.

An estimated 2.5–3 billion persons live in tropical and subtropical regions where dengue virus (DENV) is transmitted (*1*–*3*). Absence of inexpensive and accurate tests to diagnose dengue makes case management, surveillance, and outbreak investigation difficult. During infection, immunoglobulin (Ig) M against DENV can often be detected ≈5 days after onset of fever (*4**–**6*). First-time (primary) DENV infections typically have a stronger and more specific IgM response than subsequent (secondary) infections, for which the IgM response is low compared with a strong IgG response. These patterns underscore the need for evaluating the performance of commercially available tests, especially for diagnosis of secondary DENV infections (*7*–*10*).

## The Study

To provide independent evaluation of dengue diagnostic tests, the United Nations International Children’s Emergency Fund/United Nations Development Programme/World Bank/World Health Organization Special Programme for Research and Training in Tropical Diseases and the Pediatric Dengue Vaccine Initiative established a network of 7 laboratories based on criteria related to dengue expertise of the principal investigator, and type, capacity, management of the laboratory. The laboratories contributed serum specimens for the evaluation panel and conducted the evaluation. The 7 laboratories were located at Mahidol University (Bangkok, Thailand), Cho Quan Hospital (Ho Chi Minh City, Vietnam), Institut Pasteur (Phnom Penh, Cambodia), University of Malaya (Kuala Lumpur, Malaysia), Centers for Disease Control and Prevention (CDC) (San Juan, PR, USA), Instituto Medicina Tropical Pedro Kouri (Havana, Cuba), and Instituto Nacional Enfermedades Virales Humanas Dr. Julio I. Maiztegui (Buenos Aires, Argentina). Laboratories at Mahidol University and CDC acted as reference laboratories by providing samples for proficiency testing among laboratories and for assembling and validating the evaluation panel.

The evaluation panel consisted of 350 well-characterized serum specimens (Table 1). Specimens positive for IgM against DENV were obtained from patients with primary and secondary infections and represented all 4 DENV serotypes. IgM levels were determined by reference standard ELISAs developed by CDC and the Armed Forces Research Institute of Medical Science (Bangkok, Thailand) (*6*,*7*). Positive samples were selected based on optical density (OD) and were weighted toward low and medium ODs. Negative control samples included serum samples from healthy persons in areas where dengue is not endemic and from patients with other flavivirus infections, febrile illness of other causes, or systemic conditions. Results were confirmed as negative for IgM antibodies against DENV by using predetermined reference standards. Additionally, 20 anti-DENV IgM-negative specimens were obtained from SeraCare Diagnostics (Milford, MA, USA). Panel specimens were coded, heat-inactivated, aliquoted, and lyophilized; 1 aliquot was retested by the reference laboratories after reconstitution.

**Table 1:** Panels used for evaluation of 350 serum samples from patients with positive and negative results for IgM to DENV*

Evaluation panel	No. samples
DENV IgM positive	
From primary infections	27
From secondary infections	154
Total positive	181†
DENV IgM negative	
DENV positive/DENV IgM negative	19
DENV IgG positive	7
Related flavivirus IgM positive	
West Nile virus positive	25
Yellow fever virus positive	4
Related flavivirus IgG positive	
West Nile virus positive	1
Yellow fever virus positive	10
St. Louis encephalitis virus positive	2
Japanese encephalitis virus positive	10
Febrile illness	
Lyme disease IgG positive	9
Malaria	31
New World hantavirus IgM positive	7
Systemic conditions	
Rheumatoid factor	6
Systemic lupus erythematosus	2
Healthy persons‡	
Negative	36
Total negative	169

*DENV, dengue virus; Ig, immunoglobulin. †No. serum samples identified as serotype specific: 26 DENV-1, 19 DENV-2, 13 DENV-3, and 7 DENV-4. Serotype was not identified for 116 samples. ‡From areas where dengue is not endemic.

Letters of interest and the evaluation protocol were sent to 20 dengue kit manufacturers. Six companies agreed to participate and provided 4 rapid diagnostic tests (RDTs) and 5 microplate ELISAs. Test characteristics are summarized in Table 2. Price per test ranged from US $3 to $15.

**Table 2:** Characteristics of 9 tests used for detection of IgM against dengue virus*

ELISAs								
Test name	Dengue IgM capture	Pathozyme dengue M		Pathozyme dengue M capture		Dengue fever virus IgM capture DxSelect		Dengue IgM capture
Company, location	Panbio Diagnostics, Windsor, Queensland, Australia	Omega Diagnostics, Alva, UK		Omega Diagnostics		Focus Diagnostics, Cypress, CA, USA		Standard Diagnostics, Kyonggi-do, South Korea
Detection method	IgM capture	Indirect IgM detection		IgM capture		IgM capture		IgM capture
Format	12 strips of 8 wells	12 strips of 8 wells		12 strips of 8 wells		12 strips of 8 wells		12 strips of 8 wells
No. tests/package	96	96		96		96		96
Antigen	Recombinant DENV 1–4	Purified DENV 2		DENV 1–4		DENV 1–4		DENV 1–4
Sample volume, μL	10	10		20		10		10
Total incubation time	130 min at 37°C	120 min at 37°C		110 min at 37°C		240 min at room temperature		130 min at 37°C
Storage conditions, °C	2–30	2–8		2–8		2–8		2–8
Rapid diagnostic tests								
Test name	Dengue duo cassette		Hapalyse dengue-M PA kit		Dengucheck WB		SD dengue IgG/IgM	
Company, location	Panbio Diagnostics		Pentax, Tokyo, Japan		Zephyr Biomedicals, Panaji, India		Standard Diagnostics	
Assay principle	Lateral flow		Particle agglutination		Lateral flow		Lateral flow	
Target antibody	IgM and IgG		IgM		IgM and IgG		IgM and IgG	
Format	Cassette		12 strips of 8 wells		Cassette		Cassette	
No. tests/package	25		96		25		25	
Antigen	Recombinant DENV 1–4		DENV 1–4		Recombinant DENV (serotype not specified)		Recombinant DENV 1–4 envelope protein	
Specimen type	Serum, plasma, or whole blood		Serum or plasma		Serum, plasma, or whole blood		Serum or plasma	
Volume of sample required, μL	10		1		5		5	
Duration of test, min	15		90		15		15–20	
Storage conditions, °C	2–30		2–8		4–30		1–30	
Additional equipment required	No		Yes (e.g., micropipette)		No		No	

*Ig, immunoglobulin; DENV, dengue virus.

Laboratories evaluated the kits for sensitivity and specificity by using the evaluation panel. For each test, kappa coefficient values were determined to assess agreement of mean sensitivity and specificity of each test with the reference standard. A test of homogeneity was used to determine extent of agreement of results among sites.

**Figure 1:**
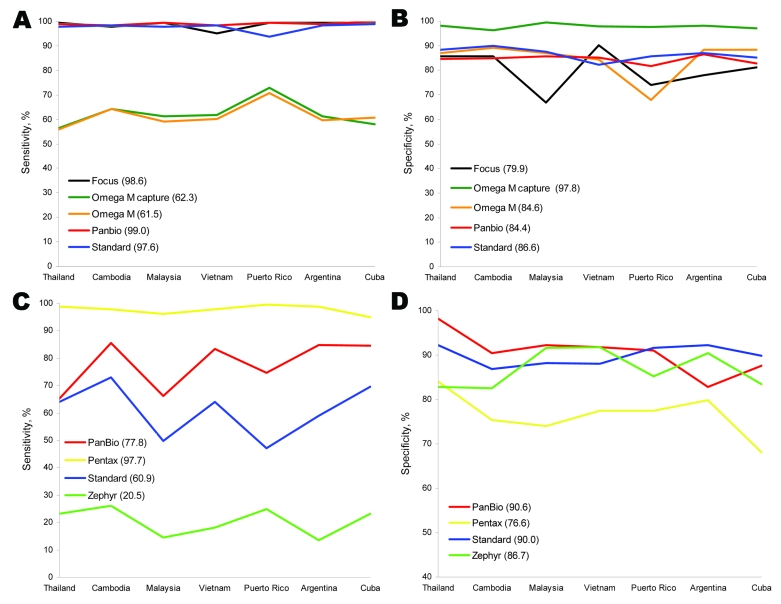
A) Sensitivity and B) specificity of 5 microplate ELISAs used at laboratories in 7 countries for detecting immunoglobulin (Ig) M against dengue virus compared with reference solid-phase IgM antibody-capture ELISAs developed by the Centers for Disease Control and Prevention (Atlanta, GA, USA) and the Armed Forces Research Institute of Medical Science (Bangkok, Thailand). Mean sensitivities and specificities for the 5 tests are shown in parentheses. C) Sensitivity and D) specificity of 4 rapid diagnostic tests used at laboratories in 7 countries for detecting IgM against dengue virus compared with solid-phase IgM antibody-capture ELISAs. Mean sensitivities and specificities for the 4 tests are shown in parentheses.

Mean sensitivities of ELISAs were 61.5%–99.0%, and specificities were 79.9%–97.8% (Figure 1, panels A and B). Tests from Panbio Diagnostics (Windsor, Queensland, Australia), Focus Diagnostics (Cypress, CA, USA), and Standard Diagnostics (Kyonggi-do, South Korea) showed significantly higher mean sensitivities (99.0%, 95% confidence interval [CI] 98.4%–99.5%; 98.6%, 95% CI 98.0%–99.2%; and 97.6%, 95% CI 96.8%–98.4%, respectively) than 2 tests from Omega Diagnostics (Alva, UK) (62.3% and 61.5%; p<0.0001 for all comparisons). The Omega Pathozyme Capture test showed significantly higher mean specificity (97.8%, 95% CI 97.0%–98.6%) than the other ELISAs (79.9%–86.6%; p≤0.02 for all comparisons). The Focus, Panbio, and Standard ELISAs showed strong agreement with the reference standard (kappa values 0.81–0.85). Kappa values for Omega kits were below the acceptable range (0.46 and 0.59). Site-to-site variation for ELISAs was not significant (homogeneity >0.05).

**Figure 2:**
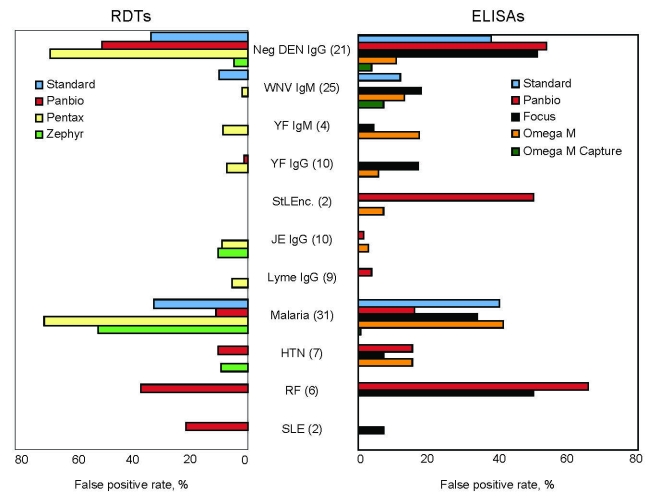
Percentages of false-positive results for 4 rapid diagnostic tests (RDTs) (left panel) and 5 microplate ELISAs (right panel). Numbers of samples tested are shown in parentheses. Neg, negative; DEN, dengue; IgG, immunoglobulin G; WNV West Nile virus; YF, yellow fever; StLEnc, St. Louis encephalitis; JE, Japanese encephalitis; Lyme, Lyme disease; HTN, New World hantavirus; RF, rheumatoid factor; SLE, systemic lupus erythematosus.

Mean sensitivities of RDTs were 20.5%–97.7%, and specificities were 76.6%–90.6% (Figure 1, panels C and D). None had an acceptable kappa value for overall performance compared with reference methods. The Pentax (Tokyo, Japan) test had significantly higher mean sensitivity (97.7%, 95% CI 96.9%–98.5%) than all other RDTs (p<0.0001 for all comparisons), but lowest mean specificity (76.6%, 95% CI 74.1%–79.0%; p<0.0001 for all comparisons) and high false-positive rates for malaria and anti-DENV IgG specimens (Figure 2). Panbio and Standard tests showed high mean specificities (90.6%, 95% CI 88.9%–92.3%, and 90.0%, 95% CI 88%.3–91.7%) with different mean sensitivities (77.8%, 95% CI 75.5%–80.1%, and 60.9%, 95% CI 58.2%–63.6%).

## Conclusions

This laboratory-based evaluation used a serum panel to determine the ability of 9 commercially available anti-DENV IgM tests to detect low levels of IgM and to determine specificity against pathogens that often cocirculate with DENV. Field trials are needed to determine the performance and utility of these tests in a local context.

Of the 5 ELISA kits evaluated, 3 (Focus, Panbio, and Standard) showed strong agreement with reference standard results and were consistent across all evaluation sites. Of concern are false-positive results shown by some tests on sera that were anti-DENV IgM negative but malaria positive, anti-DENV IgG positive, or rheumatoid factor positive. The laboratory at Mahidol University also tested the kits against 12 serum samples from patients with leptospirosis. The Panbio ELISA showed cross-reactivity with 58% of these samples, and the Focus ELISA showed cross-reactivity with 25%. Further studies are needed to elucidate the cause of this cross-reactivity.

Technicians were asked to score tests’ user-friendliness. All RDTs scored higher than ELISAs, and the Panbio RDT scored highest.

Limitations of anti-DENV IgM tests include their inability to identify the infecting DENV type and potential antibody cross-reactivity with other flaviviruses (*11**,**12*). However, cross-reactivity to related viruses did not appear to be a problem with these tests. IgM tests can be useful for surveillance and support diagnosis of DENV infection in conjunction with clinical symptoms, medical history, and other epidemiologic information (*13*). Because IgM persists for >60 days, IgM assays should not be used in dengue-endemic countries as confirmatory tests for current illness. Presence of IgM indicates that a dengue infection has occurred in the past 2–3 months.

This evaluation has several limitations. Test performance was compared with reference laboratory assay results, which may be less sensitive than commercial assays, leading to some results being misclassified as false positive. Specificity of these tests may be higher in a field setting than in this evaluation because not all potential causes of false-positive results would be present. The panel consisted of a high proportion of specimens from persons with secondary DENV infections. Thus, the panel was weighted toward lower anti-DENV IgM levels. However, this feature reflects the situation in most dengue-endemic countries. Thus, tests that performed well against this panel could be expected to perform well in these diagnostic settings. We could not comprehensively evaluate whether the kits could detect primary infections with all 4 DENVs because all DENV types were not represented in the panel.

Data from this evaluation have been provided to the manufacturers and WHO member states. On the basis of these results, ELISAs from Focus, Panbio, and Standard Diagnostics will be included in the WHO Bulk Procurement Scheme. Technical discussions are ongoing to determine how tests might be improved to accelerate availability of useful methods for dengue case management, surveillance, and disease control.

## References

[1] World Health Organization 2008. Dengue and dengue haemorrhagic fever. Factsheet no. 117, revised May 2008 [cited 2008 Jun 5]. Available from http://www.who.int/mediacentre/factsheets/fs117/en

[2] Gubler D. Dengue and dengue hemorrhagic fever: its history and resurgence as a global public health problem. In: Gubler DJ, Kuno G, editors. Dengue and dengue hemorrhagic fever. Cambridge (MA): CAB International; 1997. p. 1–22.

[3] Halstead SB. Dengue. Lancet. 2007;370:1644–52. 10.1016/S0140-6736(07)61687-0 17993365

[4] Guzman MG, Kouri G. Dengue diagnosis, advances and challenges. Int J Infect Dis. 2004;8:69–80. 10.1016/j.ijid.2003.03.003 14732325

[5] Vaughn DW, Green S, Kalayanarooj S, Innis BL, Nimmannitya S, Suntayakorn S, Dengue in the early febrile phase: viremia and antibody responses. J Infect Dis. 1997;176:322–30. 9237696 10.1086/514048

[6] Innis BL, Nisalak A, Nimmannitya S, Kusalerdchariya S, Chongswasdi V, Suntayakorn S, An enzyme-linked immunosorbent assay to characterize dengue infections where dengue and Japanese encephalitis co-circulate. Am J Trop Med Hyg. 1989;40:418–27. 2540664 10.4269/ajtmh.1989.40.418

[7] Burke DS, Nisalak AA, Ussery MM. Antibody capture immunoassay detection of Japanese encephalitis virus immunoglobulin M and G antibodies in cerebrospinal fluid. J Clin Microbiol. 1982;16:1034–42. 7161371 10.1128/jcm.16.6.1034-1042.1982 PMC272535

[8] Miagostovich MP, Nogueira RM, dos Santos FB, Schatzmayr HG, Araujo ES, Vorndam V. Evaluation of an IgG enzyme-linked immunosorbent assay for dengue diagnosis. J Clin Virol. 1999;14:183–9. 10.1016/S1386-6532(99)00059-1 10614855

[9] Groen J, Koraka P, Velzing J, Copra C, Osterhaus AD. Evaluation of six immunoassays for detection of dengue virus–specific immunoglobulin M and G antibodies. Clin Diagn Lab Immunol. 2000;7:867–71. 10.1128/CDLI.7.6.867-871.2000 11063489 PMC95976

[10] Kit Lam S, Lan Ew C, Mitchell JL, Cuzzubo AJ, Devine PL. Evaluation of capture screening enzyme-linked immunosorbent assay for combined determination of immunoglobulin M and G antibodies. Clin Diagn Lab Immunol. 2000;7:850–2. 10.1128/CDLI.7.5.850-852.2000 10973469 PMC95970

[11] Blacksell SD, Newton PN, Bell D, Kelley J, Mammen MP, Vaughn DW, The comparative accuracy of 8 rapid immunochromatographic assays for the diagnosis of acute dengue virus infection. Clin Infect Dis. 2006;42:1127–34. 10.1086/501358 16575730

[12] Vázquez S, Valdés O, Pupo M, Delgado I, Alvarez M, Pelegrino JL, MAC-ELISA and ELISA inhibition methods for detection of antibodies after yellow fever vaccination. J Virol Methods. 2003;110:179–84. 10.1016/S0166-0934(03)00128-9 12798246

[13] Wichmann O, Gascon J, Schunk M, Puente S, Siikamaki H, Gjørup I, European Network on Surveillance of Imported Infectious Diseases. Severe dengue virus infection in travelers: risk factors and laboratory indicators. J Infect Dis. 2007;195:1089–96. 10.1086/512680 17357044

